# The Community-Reinforcement Approach

**Published:** 1999

**Authors:** William R. Miller, Robert J. Meyers, Susanne Hiller-Sturmhöfel

**Affiliations:** William R. Miller, Ph.D., is director of research and Robert J. Meyers, M.S., is a senior research scientist at the Center on Alcoholism, Substance Abuse, and Addiction, University of New Mexico, Albuquerque, New Mexico. Susanne Hiller-Sturmhöfel, Ph.D., is a science editor of Alcohol Research & Health

**Keywords:** AODU (alcohol and other drug use) treatment method, reinforcement, AOD (alcohol and other drug) abstinence, motivation, AOD use pattern, AODD (alcohol and other drug dependence) recovery, treatment outcome, cessation of AODU, professional client relations, family therapy, motivational interviewing, spouse or significant other, literature review

## Abstract

The community-reinforcement approach (CRA) is an alcoholism treatment approach that aims to achieve abstinence by eliminating positive reinforcement for drinking and enhancing positive reinforcement for sobriety. CRA integrates several treatment components, including building the client’s motivation to quit drinking, helping the client initiate sobriety, analyzing the client’s drinking pattern, increasing positive reinforcement, learning new coping behaviors, and involving significant others in the recovery process. These components can be adjusted to the individual client’s needs to achieve optimal treatment outcome. In addition, treatment outcome can be influenced by factors such as therapist style and initial treatment intensity. Several studies have provided evidence for CRA’s effectiveness in achieving abstinence. Furthermore, CRA has been successfully integrated with a variety of other treatment approaches, such as family therapy and motivational interviewing, and has been tested in the treatment of other drug abuse.

In nearly every review of alcohol treatment outcome research, the community-reinforcement approach (CRA) is listed among approaches with the strongest scientific evidence of efficacy. Yet many clinicians who treat alcohol problems have never heard of it, despite the fact that the first clinical trial of CRA was published over a quarter of a century ago—an example of the continuing gap between research and practice.

The underlying philosophy of CRA is disarmingly simple: In order to overcome alcohol problems, it is important to rearrange the person’s life so that abstinence is more rewarding than drinking. The use of alcohol as well as other drugs can be highly reinforcing: The user experiences effects that motivate him or her to continue drinking, which can lead to alcohol dependence.

What then would make a dependent drinker want to give up drinking? One common approach is to “turn up the pain”—that is, to confront the person with unpleasant and costly consequences of drinking. This approach attempts to render drinking less attractive, and can include aversion therapies, pharmacotherapy with the medication disulfiram, confrontational counseling, and infliction of negative consequences (i.e., punishment). Such negative approaches, however, frequently have been found to be ineffective in decreasing drinking and alcohol problems (Miller et al. 1998). Even seasoned clinicians are often amazed at how much adversity an alcoholic will endure in order to continue drinking. In fact, it has been said that if punishment worked, there would be no alcoholics.

CRA takes a different approach to overcoming alcohol problems, one that is based on providing incentives to stop drinking rather than punishment for continued drinking. To that end, client, therapist, and significant others work together to change the drinker’s lifestyle (e.g., his or her social support system and activities) so that abstinence becomes more rewarding than drinking. Since its introduction by Hunt and Azrin in 1973, CRA treatment has evolved considerably, and the clientele has expanded to include spouses of alcoholics and users of drugs other than alcohol. This article summarizes the components of CRA as well as factors influencing its effectiveness. In addition, the article briefly reviews clinical studies demonstrating CRA’s efficacy in treating clients with alcohol and other drug (AOD) problems.

## What is CRA?

To provide an alcoholic with the incentive to quit drinking, CRA has the following two major goals:

- Elimination of positive reinforcement for drinking
- Enhancement of positive reinforcement for sobriety.

To achieve those goals, CRA therapists combine a variety of treatment strategies, such as increasing the client’s motivation to stop drinking, initiating a trial period of sobriety, performing a functional analysis of the client’s drinking behavior, increasing positive reinforcement through various measures, rehearsing new coping behaviors, and involving the client’s significant others. Other factors, such as therapist style and initial treatment intensity, also may influence the client’s outcome. These treatment components and treatment-related factors are described in the following sections.

### Building Motivation

The initial step in CRA generally is an exploration of the client’s motivations for change. Particularly in early versions of CRA, this process involved the identification of positive reinforcers (e.g., praise and shared pleasant events) that could serve as effective incentives for the client to change his or her behavior. The CRA therapist also reviews with the client the current and future negative consequences of the client’s drinking patterns. For example, the therapist may offer an “inconvenience review checklist”—a list of frequent negative consequences of drinking, such as medical problems, marital problems, or difficulties at work. The client then checks all those negative consequences that apply to his or her current situation or are likely to occur in the future. This assessment can be conducted in an empathic motivational interviewing style rather than a confrontational style (Miller and Rollnick 1991), thereby encouraging the client, rather than the therapist, to voice the advantages of change and the disadvantages of his or her current drinking.

### Initiating Sobriety

Once the client has identified factors that provide the motivation to change his or her drinking behavior, the therapist moves on to setting goals for achieving abstinence. Because many clients are reluctant to commit themselves to immediate total and permanent abstinence, a procedure called sobriety sampling can be helpful. This procedure uses various counseling strategies to negotiate intermediate goals, such as a trial period of sobriety (see Miller and Page 1991). For example, the therapist may encourage the client to try not drinking for 1 month, to see how it feels and to learn more about the ways in which he or she has been depending on alcohol. Sanchez-Craig and colleagues (1984) found that clients who explicitly were given a choice about a trial period of abstinence were more likely to abstain than were clients who were given a firm prescription for abstinence.

### Analyzing Drinking Patterns

CRA involves a thorough functional analysis of the client’s drinking patterns. This analysis helps identify situations in which drinking is most likely to occur (i.e., high-risk situations) as well as positive consequences of alcohol consumption that may have reinforced drinking in the past. This step, which is often underemphasized in cognitive-behavioral therapy, is useful in individualizing treatment and in determining specific treatment components, or modules, that are most likely to be successful for a particular client.

### Increasing Positive Reinforcement

Once the analysis of the client’s drinking patterns is completed, both the client and therapist select appropriate modules from a menu of treatment procedures to address the client’s individual needs. Many of these treatment modules focus on increasing the client’s sources of positive reinforcement that are unrelated to drinking. For example, as people become increasingly dependent on alcohol, their range of non-drinking activities (e.g., hobbies, sports, and social involvement) narrows substantially, resulting in increasing isolation. Consequently, an important component of recovery for the drinker is to reverse this isolation process by becoming involved with other nondrinking people and by increasing the range of enjoyable activities that do not involve drinking.

Several treatment modules can help in this process. For example, social and recreational counseling is used to help the client choose positive activities to fill time that was previously consumed by drinking and recuperating from its effects. If the client cannot easily decide on such activities, an approach called activity sampling can encourage him or her to try out or renew various activities that might be, or once were, fun and rewarding. For this strategy, the therapist and client schedule activities that the client will try between counseling sessions and plan where, when, how, and with whom the client will participate in those activities. Those plans emphasize activities that bring the client into contact with other people in nondrinking contexts. Such activities might include involvement in a church, attendance of 12-step meetings or classes, participation in common-interest clubs (e.g., sports clubs), visits to alcohol-free establishments, or participation in volunteer programs. The choice of programs is tailored to the client’s personal interests to ensure that the client experiences the activities as positive reinforcers.

Other components of CRA are designed to help clients organize not only their leisure activities but also, if necessary, their regular daily lives. For example, a component called access counseling addresses practical barriers, such as the lack of information sources and means of communication, that stand between the client and those activities that provide positive reinforcement. Thus, access counseling assists the client in obtaining everyday necessities, such as a telephone, a newspaper, a place to live, or a job. Another approach to helping clients find rewarding work involves job club procedures (e.g., interview skills training and résumé development), which have been shown to be successful even for difficult-to-employ people (Azrin and Besalel 1980). The common goal of all these CRA treatment modules is to make the client’s alcohol-free life more rewarding and affirming and to re-engage the client in his or her community.

### Behavior Rehearsal

CRA therapists do not just talk about new behavior; instead, they have clients actually practice new coping skills, particularly those involving interpersonal communication, during the counseling sessions. For example, a therapist may first demonstrate the new behavior (e.g., drink refusal or assertive communication), then reverse roles and guide the client in practicing the new skill. Again, the therapist gives praise for any and all steps in the right direction.

### Involving Significant Others

Because CRA emphasizes change not only in the client’s behavior but also in his or her social environment, this treatment approach emphasizes and encourages, whenever possible, the cooperation of other people who are close and significant to the drinker. Significant others, particularly those who live with a drinker, can be helpful in identifying the social context of the client’s drinking behavior and in supporting change in that behavior. Consequently, even early versions of CRA included brief relationship counseling (Hunt and Azrin 1973). Rather than providing protracted marital therapy, this counseling offers practical skills training to improve positive communication and reinforcement between the client and his or her significant other, reduce aversive communication (e.g., arguments), and facilitate the negotiation of specific changes in the drinker’s behavior (Meyers and Smith 1995). In addition, CRA therapists may coach significant others on how to avoid inadvertent reinforcement of drinking (sometimes called “enabling”) and increase positive reinforcement for sobriety—for example, by spending time with the drinker when he or she is sober and withdrawing attention when he or she is drinking.

### Factors Influencing CRA Effectiveness

In addition to the treatment components previously described, several factors related to treatment delivery may influence treatment effectiveness and, consequently, the patient’s outcome. Two of those factors are therapist style and initial treatment intensity.

#### Therapist Style

An important aspect of CRA that is sometimes underemphasized is the therapeutic style with which this treatment approach is delivered. An optimal CRA therapist is consistently positive, energetic, optimistic, supportive, and enthusiastic. Any and all signs of progress, no matter how small—even the client just showing up for a counseling session—are recognized and praised. CRA counseling is provided in a personal, engaging style, not in the form of a businesslike negotiation or impersonal education. With a therapist who successfully executes this counseling approach, clients look forward to coming back for future sessions and leave those sessions feeling hopeful and good about themselves.

Although many therapists can deliver CRA, some clinicians might find this approach easier to adopt than will other clinicians. For example, therapists with generally optimistic or enthusiastic personalities might be best suited for CRA. In contrast, therapists who have been trained to use a relatively confrontational approach in order to break down denial may find the CRA approach more difficult to practice.

#### Initial Treatment Intensity

Another characteristic of CRA that may contribute to the success of this approach is its “jump-start” quality. Ideally, a client who is ready for change can schedule an appointment for the same or following day, rather than being placed on a waiting list for 1 or more months. In addition, during the initial treatment phase, counseling sessions may be scheduled more frequently than once per week. The intervals between sessions can then be extended as the client’s abstinence becomes more stable.

Finally, CRA can involve procedures to initiate abstinence immediately. For example, in some cases the client can be evaluated right away as to whether he or she is a candidate for taking disulfiram, an agent that induces unpleasant effects (e.g., nausea and vomiting) after alcohol consumption and is used to discourage drinking. In those cases, a medical staff member of the treatment facility can promptly issue and fill a disulfiram prescription, and the client can take the first dose in the therapist’s presence. If a concerned significant other is willing to help the client, he or she can be trained along with the client in procedures to ensure that the client takes the medication regularly. This process also can be used to promote patient compliance with other medication regimens.

## Evidence For CRA’S Efficacy

During the past 25 years, numerous studies have demonstrated the efficacy of CRA in the treatment of alcoholism. In the first evaluation, Hunt and Azrin (1973) compared CRA with traditional disease-model treatment^1^ for alcohol-dependent people receiving inpatient treatment. In that study, the patients who received CRA fared much better than did the patients who received traditional treatment—in fact, almost no overlap existed in the distribution of the two groups on several outcome measures at followup. The CRA clients drank substantially less and less often, had fewer institutionalized days and more days of employment, and exhibited greater social stability compared with patients who were treated with the traditional approach.

Additional improvements to CRA, such as monitored disulfiram administration, mood monitoring, and spousal involvement, further increased the difference in outcome between patients who received CRA and those who received traditional treatment (Azrin 1976). For example, with these improvements, drinking days in the CRA group dropped to 2 percent of all days during a 6-month followup period compared with 55 percent of all days in the standard treatment group.

Whereas those initial studies were conducted in an inpatient setting, CRA subsequently was shortened and adapted for use in outpatient settings (e.g., by instituting immediate disulfiram administration). The modified CRA approach also was considerably more effective than traditional outpatient treatment mirroring the Minnesota model (Azrin et al. 1982). As in previous studies, CRA clients showed substantially increased rates of abstinence and employment and less institutionalization and incarceration.

In another study, researchers assessed the effectiveness of a social intervention consistent with CRA. They provided an alcohol-free club where clients could socialize and have fun without drinking. Clients given access to this club evidenced better outcomes than did clients without such access (Mallams et al. 1982). Because these studies employed scientifically sound methodology, they provided strong evidence for the effectiveness of CRA.

Other evaluations of CRA’s effectiveness have been conducted at the University of New Mexico’s Center on Alcoholism, Substance Abuse, and Addictions (CASAA). In one outpatient treatment study (Meyers and Miller in press), CRA was found to be more successful in suppressing drinking than was a traditional disease-model counseling treatment approach.

After Meyers and Smith (1995) published the first manual delineating the components of CRA for therapists treating patients with alcohol problems, CASAA researchers conducted a study on CRA’s efficacy among homeless alcohol-dependent men and women at a large day shelter. The study found that compared with the standard 12-step-oriented group therapy provided at the shelter, CRA, when implemented as described in the manual, resulted in significantly improved outcomes during the 1-year followup period (Smith et al. 1998). As in previous studies, alcohol consumption in the CRA group was almost completely suppressed during 1 year of followup. In contrast, patients in the standard care group reported drinking on about 40 percent of the days as well as high levels of intoxication.

### CRA as Family Therapy

In recent years, CRA also has been integrated into a unilateral family therapy (FT) approach in which the person seeking help is not the drinker (who refuses to get treatment) but a concerned spouse or other family member—resulting in the community reinforcement and family training (CRAFT) approach (Meyers and Smith 1997). The CRAFT treatment approach is based on studies demonstrating that the involvement of family members can help initiate and promote the treatment of people with alcohol problems (Sisson and Azrin 1986).

Without the drinker present, the CRAFT therapist works with the family member to change the drinker’s social environment in a way that removes inadvertent reinforcement for drinking and instead reinforces abstinence. The therapist also helps the family member prepare for the next opportunity when the drinker may respond favorably to an offer of help and support and may be willing to enter treatment.

A recently completed clinical trial funded by the National Institute on Alcohol Abuse and Alcoholism evaluated the efficacy of CRAFT (Miller et al. in press). In that study, 64 percent of the clients who received CRAFT counseling succeeded in recruiting their loved one into treatment following an average of four to five counseling sessions. In contrast, two traditional methods for engaging unmotivated problem drinkers into treatment—the Johnson Institute intervention^2^ and counseling to engage in Al-Anon—resulted in significantly lower proportions of significant others (30 percent and 13 percent, respectively) motivating their loved ones to enter treatment. In a parallel study sponsored by the National Institute on Drug Abuse that focused on abusers of other drugs, family members receiving CRAFT successfully engaged 74 percent of initially unmotivated drug users in treatment (Meyers et al. 1999).

### CRA in the Treatment of Other Drug Abuse

CRA also has been used in the treatment of other drug abuse and dependence. For example, researchers at CASAA conducted a trial in which heroin addicts receiving methadone maintenance therapy were randomly assigned to CRA or standard treatment approaches. Although both CRA and the traditional approaches resulted in good treatment outcomes in this study, CRA was associated with a modest but statistically significant advantage over the standard care approaches (Abbott et al. 1998). Furthermore, researchers studying the treatment of cocaine addicts found substantially better outcomes for clients who received CRA combined with positive reinforcement in the form of monetary vouchers issued when the clients tested drug free compared with clients who participated in an outpatient 12-step counseling program (Higgins et al. 1991).

## Can CRA Be Used in Ordinary Treatment Settings?

CRA has sometimes been delivered in relatively expensive ways (e.g., in inpatient programs, through home visits, and in combination with vouchers). However, CRA is also amenable to and effective in the typical outpatient treatment context, in which the client is seen weekly at a clinic. Furthermore, in outpatient studies that demonstrated good treatment outcomes with CRA, alcohol-dependent patients received an average of five to eight CRA sessions (e.g., Azrin et al. 1982). Similarly, the study by Miller and colleagues (in press) demonstrated that approximately five CRAFT sessions with a concerned significant other frequently resulted in the drinker’s entry into treatment. This treatment duration is well within the guidelines of most managed care systems.

Although CRA is based on a comprehensive treatment philosophy, its procedures generally are familiar to clinicians who have been trained in cognitive-behavioral treatment approaches. For example, CRA involves a functional analysis and the individualized application of specific components chosen from a menu of problem-solving procedures. Furthermore, CRA can be combined with other treatment methods. For example, at CASAA, CRA has recently been combined with motivational interviewing to form an integrated treatment. Similarly, CRA is consistent with involvement in 12-step programs. Finally, combinations of CRA and other treatment approaches can be tailored to address the needs of particular client populations (for an example of such an approach targeted to a specific population, see sidebar, p. 121).

## Summary

CRA is a comprehensive, individualized treatment approach designed to initiate changes in both lifestyle and social environment that will support a client’s long-term sobriety. CRA focuses on finding and using the client’s own intrinsic reinforcers in the community and is based on a flexible treatment approach with an underlying philosophy of positive reinforcement. Those characteristics make CRA (with certain modifications) applicable to a wide range of client populations.

Numerous clinical trials have found CRA to be effective in treating AOD abuse and dependence and in helping relatives recruit their loved ones into AOD-abuse treatment. The trials were conducted in a variety of geographic regions, treatment settings (e.g., inpatient and outpatient), and individual and family therapy approaches. Furthermore, the clients in those studies suffered from various AOD-related problems and included homeless people as well as people of different ethnic or cultural backgrounds. Consistently, CRA was more effective than the traditional approaches with which it was compared or to which it had been added. Because the scope and duration of CRA are compatible with the guidelines of most managed care services, this approach may play an increasingly important role in the treatment of people with alcoholism.

**CRA and Special Populations**

**Figure:**
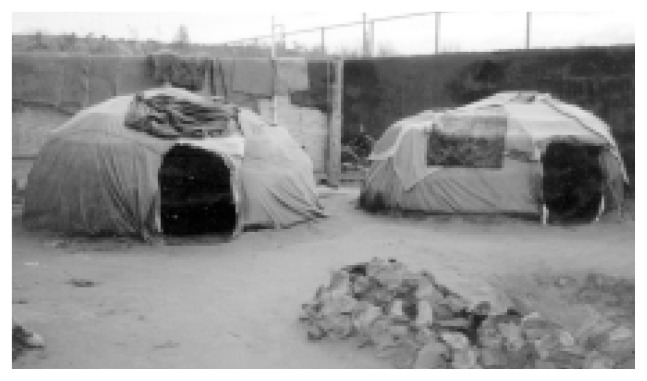

The community-reinforcement approach (CRA) is a highly flexible treatment approach that allows therapists and clients to choose from an extensive menu of treatment options to meet the specific needs of the client. This flexibility also enables CRA to be adapted easily to client populations with special needs, such as ethnic or cultural minorities. For example, CRA has been adapted creatively at the Na’nizhoozhi Center, Inc. (NCI) in Gallup, New Mexico, a treatment facility that primarily serves the *Dine’* (Navajo) Native Americans. The NCI staff has developed an intensive, 16-day residential program for alcohol-dependent Native Americans who have not responded to treatment programs based on the Minnesota model, which emphasizes a loss-of-control disease methodology and a 12-step approach to recovery.

Among the *Dine’*, clan ties remain strong even when the trust between the drinker and his or her family has been broken repeatedly. Accordingly, when treating clients from this cultural group, alcoholism treatment professionals should work with both the family and community networks using traditional Native American ceremonies and extended clan ties. The NCI program connects or reconnects the clients with Native American spirituality through the *Hiina’ah Bits’os* (Eagle Plume) Society. For example, traditional practices, such as the talking circle (i.e., the passing of an object that designates who is speaking while all others listen) and the sacred use of tobacco, are integrated into the treatment program, replacing alcohol with the *Dine’* way of seeking harmony with all of creation (i.e., “walking in beauty”). A special compound built adjacent to the Na’nizhoozhi Center includes ceremonial grounds, tepees, and sweat lodges.

For many NCI clients, the path into this program has been long and painful. Most clients are unemployed, destitute, hopeless, physically ill, and depressed after multiple treatment failures. Researchers have begun to evaluate the effectiveness of the modified CRA approach practiced at the NCI in this challenging patient population. Preliminary results indicate that at the 6-month followup, a substantial portion of clients have achieved continuous abstinence and many other clients are free of alcohol-related problems, despite occasional drinking, or have improved considerably even if they have experienced some ongoing problems. These initial indications of effectiveness bear witness to the *Hiina’ah Bits’os* Society’s motto, “Against all odds, we walk in beauty.” *—William R. Miller and Robert J. Meyers with Susanne Hiller-Sturmhöfel*

**Figure:**
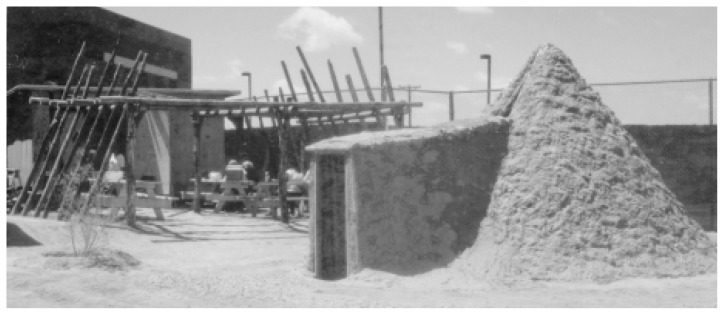

## Footnotes

1 Traditional disease-model treatment is similar to the Minnesota model, which posits that alcoholism is a disease characterized by loss of control over drinking and which emphasizes a 12-step approach to recovery.

2 The Johnson Institute intervention entails five therapy sessions that prepare the client and his or her family members for a family confrontation meeting.
